# Undernutrition is associated with perturbations in T cell-, B cell-, monocyte- and dendritic cell- subsets in latent *Mycobacterium tuberculosis* infection

**DOI:** 10.1371/journal.pone.0225611

**Published:** 2019-12-10

**Authors:** Anuradha Rajamanickam, Saravanan Munisankar, Chandra Kumar Dolla, Subash Babu

**Affiliations:** 1 National Institute of Health-NIRT-International Center for Excellence in Research, Chennai, India; 2 National Institute for Research in Tuberculosis, Chennai, India; 3 Laboratory of Parasitic Diseases, National Institute of Allergy and Infectious Diseases, National Institutes of Health, Bethesda, Maryland, United States of America; Rutgers Biomedical and Health Sciences, UNITED STATES

## Abstract

Undernutrition, as described by low body mass index (BMI), is a foremost risk factor for the progression of active Tuberculosis (TB). Undernutrition is also known to impact the baseline frequencies of innate and adaptive immune cells in animal models. To verify whether undernutrition has any influence on the baseline frequencies of immune cells in latent *Mycobacterium tuberculosis* infection (LTBI), we examined the frequencies of T cell-, B cell, monocyte- and dendritic cell (DC)- subsets in individuals with LTBI and low BMI (LBMI) and contrasted them with LTBI and normal BMI (NBMI) groups. LBMI was characterized by decreased frequencies and absolute cell counts of T cells, B cells and NK cells in comparison with NBMI. LBMI individuals demonstrated significantly enhanced frequencies of naïve and effector CD4^+^ and CD8^+^ T cells and significantly decreased frequencies of central memory, effector memory CD4^+^ and CD8^+^ T cells and regulatory T cells. Among B cell subsets, LBMI individuals demonstrated significantly diminished frequencies of naïve, immature, classical memory, activated memory, atypical memory and plasma cells. In addition, LBMI individuals showed significantly decreased frequencies of classical monocytes, myeloid DCs and plasmacytoid DCs and significantly increased frequencies of intermediate and non-classical monocytes and myeloid derived suppressor cells. BMI exhibited a positive correlation with B cell and NK cell counts. Our data, therefore, demonstrates that coexistent undernutrition in LTBI is characterized by the occurrence of a significant modulation in the frequency of innate and adaptive immune cell subsets.

## Introduction

Globally, Tuberculosis (TB) continues as the foremost reason for infection related illness and death. In 2017, the World Health Organization reported 10.4 million TB cases with 1.7 million deaths annually (World Health Organization. Global tuberculosis report, 2018. WHO Geneva, Switzerland: who.int, 2018). The manifestation of TB infection and disease range from latent infection to pulmonary or extrapulmonary disease. Individuals with Latent tuberculosis infection (LTBI) are asymptomatic and have a recall immune response to mycobacterial antigens. Globally, approximately 23% of the population are with LTBI [1]. Among individuals with LTBI, only about 5 to 10% develop active TB during their life time and this conversion occurs due to breakdown in the protective immune mechanism [2].

Both nutrition and immunity are strongly interlinked. Innate and the adaptive immune systems are influenced by nutritional status and these immune cells have a role in nutritional immunology. Phagocytosis, T cell numbers and cell-proliferation response to mitogens are affected due to undernutrition [3,4]. Nutritionally compromised individuals, who had vaccination, also exhibited diminished specific antibody titers [5]. Undernutrition dampens the cell-mediated immunity and predisposes individuals to become more vulnerable to active TB disease [4, 6, 7]. Several developing countries have high TB burdens concomitant with undernutrition. Undernutrition has the highest population attributable fraction (27%) of any risk factor in many countries with the highest TB burden [8–10].

Nonetheless, in humans, the functions of innate and adaptive immune cells in undernourished individuals with LTBI have not been explored in detail. Very few studies have shown data on the immunological mechanism of predisposition from latent to active TB disease. We postulate that undernutrition could diminish the cellular responses in LTBI and thus weaken the immune system and which in turn cause individuals with LTBI to be more prone to active TB disease. To study the effect of undernutrition on LTBI, we compared the frequencies of T cell-, B cell-, monocyte- and dendritic cell (DC)- subsets between LTBI with low BMI (LBMI) group and LTBI with normal BMI (NBMI) group.

## Materials and methods

### Ethics statement

The study protocol was approved by Institutional Review Board of the National Institute of Research in Tuberculosis, Chennai, India (approval no. NCT00375583 and NCT00001230) and as part of the natural history protocol, informed written consent was taken from all study participants.

### Study population

We enrolled 60 study participants with LTBI, with 30 participants with LBMI and 30 participants with NBMI between 2015 and 2018 (Table 1). All the participants were residents of rural villages of Kanchipuram District, Tamil Nadu, South India with an age range from 18 to 65 years. These study participants were all enrolled from a rural population by screening of individuals for BMI and LTBI. We screened a total of 200 participants with LTBI to recruit 30 with LBMI and 30 matched controls (for age and sex) with NBMI. The flow chart is given in S1 Fig.

**Table 1:** Demographic profile of the study population.

Study demographics	LBMI	NBMI	p value
	n = 30	n = 30	
M/F	18/12	18/12	0.9837
Age	39 (24–60)	41 (24–60)	0.9652
Body mass index kg/m^2^	16.7 (14.8–18.3)	22 (19.2–24.1)	0.0008
Albumin (g/dl)	2.9 (2.5–3.3)	4.1 (3.6–4.9)	0.0007
Random Blood Glucose (mg/dl)	88.3 (64–109)	93.6 (71–180)	0.2462
HbA1c (%)	5.4 (4.8–6.2)	5.7 (4.6–6.1)	0.3676
Urea (mg/dl)	20.8 (9–38)	20.2 (12–36)	0.3659
Creatinine (mg/dl)	0.78 (0.3–1)	0.73 (0.5–1)	0.5070
ALT (U/L)	18.9 (8–98)	21.8 (7–45)	0.1625
AST (U/L)	24.6 (13–69)	25.1 (13–53)	0.6382

The values depicted as geometric mean and range for sex and age depicted in median and range. **LBMI** denotes to study participants with low BMI with LTBI, **NBMI** denotes to study participants with normal BMI with LTBI. p values calculated using Mann-Whitney test.

### Study procedures

LTBI was detected as those who were positive for both tuberculin skin test (TST) and Quantiferon TB Gold in Tube test (QFT), with no indications of active TB, no history of earlier TB disease, and normal chest radiographs. TST was performed using 2 tuberculin units of tuberculin purified protein derivative (PPD) RT 23 SSI (Serum Statens Institute). Based on previously determined cut off norms for South India, a positive skin test was described as an induration of at least 12 mm in diameter [11]. QFT was done by following the manufacturer’s instructions (Qiagen). Anthropometric measurements, consisting of height, weight, and waist circumference (all measured in triplicate), and bio- chemical parameters, including plasma glucose, serum albumin, urea, creatinine, alanine aminotransferase (ALT), aspartate aminotransferase (AST), and HbA1c levels were measured using normalised methods. Low and normal BMIs were described based on the American Heart Association/American College of Cardiology guidelines (LBMI, <18.5 kg/m2; and NBMI, between 18.5 and 24.9 kg/m2). Additionally, undernutrition was confirmed using low serum albumin (<3.4 g/dl) in all the low-BMI individuals. Low BMI individuals with normal albumin were excluded. All participants who were recruited for the study were negative for diabetes, HIV and parasitic infections. Diabetes was defined as an glycated haemoglobin (HbA1c) reading of 6.5% or greater and a random blood glucose of >200 mg/dl, according to the American Diabetes Association criteria. Parasitic infections were ruled out by stool microscopy and serology. This study population was the same as the population used for our previous study on chemokine analysis [12].

### *Ex vivo* analysis

An AcT5 Diff hematology analyser (Beckman Coulter) was used to measure the Leukocyte counts and differential counts for all the study participants. The antibodies used for this study were from BD Biosciences (San Jose, CA), BD Pharmingen (San Diego, CA), eBiosciences (San Diego, CA), or R&D Systems (Minneapolis, MN). To the Trucount tube, 50ul of whole blood was added and TBNK (BD multiset) monoclonal antibody cocktail was added to the whole blood. Next, the tubes were incubated for 20 minutes at room temperature and then 450ul of FACS lysing solution was added and incubated in dark for 60 minutes (lyse no wash) and acquired in FACSCanto II clinical software. *Ex-vivo* phenotyping was done on all 60 study participants using whole blood. Concisely, 250ul aliquots of whole blood was added to a cocktail of monoclonal antibodies specific for different immune cell types. T cell phenotyping was done using antibodies directed against CD45-Peridinin chlorophyll protein (PerCP; clone 2D1, BD), CD3-AmCyan (clone SK7; BD), CD4-phycoerythrin (PE) Cy7 (clone SK3; BD), CD8-allophycocyanin (APC) H7 (clone SK1; BD), CD45RA-Pacific Blue (clone H1100; Biolegend, Cambridge, UK), and CCR7-FITC (clone 3D12; eBiosciences). Naive cells were defined as CD45RA^+^ CCR7^+^, central memory cells as CD45RA^-^ CCR7^+^, effector memory cells as CD45RA^-^CCR7^-^ and effector cells as CD45RA^-^ CCR7^-^. Regulatory T cell phenotyping was done using CD25 (clone M-A251; BD), CD127 (clone eBioRDRS; eBiosciences, San Diego, CA), Foxp3 (clone 236A/E7, eBiosciences) and regulatory T cells were defined as CD4^+^ CD25^+^ Foxp3^+^ CD127dim. B cell phenotyping was done using antibodies directed against CD45-PerCP (clone 2D1, BD), CD19-Pacific Blue (clone H1B19; Biolegend) CD27-APC-Cy7 (clone M-T271; BD), CD21-FITC (clone B-ly4; BD) CD20-PE (clone 2H7; BD) and CD10-APC (clone H110a; BD). Naive B cells were defined as CD45^+^ CD19^+^ CD21^+^ CD27^-^; classical memory B cells as CD45^+^ CD19^+^ CD21^+^ CD27^+^; activated memory B cells as CD45^+^ CD19^+^ CD21^-^ CD27^+^; atypical memory B cells as CD45^+^ CD19^+^ CD21^-^CD27^-^; immature B cells as CD45^+^ CD19^+^ CD21^+^ CD10^+^; and plasma cells as CD45^+^ CD19^+^ CD21^-^ CD20^-^. Monocyte phenotyping was done using antibodies directed against CD45-PerCP (clone 2D1; BD), CD14-Pacific Blue (clone M5E2; Biolegend) HLA-DR-PE-Cy7 (clone L243; BD) and CD16-APC- Cy7 (clone 3G8; BD). Classical monocytes were defined as CD45^+^ HLA-DR^+^ CD14^hi^ CD16^-^; intermediate monocytes as CD45^+^ HLA-DR^+^ CD14^hi^ CD16^dim^ and non-classical monocytes were defined as CD45^+^ HLA- DR^+^CD14^dim^CD16^hi^. Phenotyping of DC was done using antibodies directed against HLA- DR, lineage cocktail (CD3, CD14, CD16, CD19, CD20, CD56) FITC (clone SJ25C1, SK7, MΦp9, L27, NCAM16.2, 3G8 BD) CD123-PE (clone 9F5; BD) CD11c- APC (clone S-HCL-3; BD). Plasmacytoid DCs were defined as (Lin–HLA-DR^+^ CD123^+^) and myeloid DCs as (Lin–HLA-DR^+^ CD11c^+^). Phenotyping of myeloid derived suppressor cells (MDSCs) was done using antibodies directed against CD45-PerCP (clone 2D1; BD), CD14-Pacific Blue (clone M5E2; Biolegend), CD33 PE (Clone P67.6 BD), HLA-DR-PE-Cy7 (clone L243; BD) and CD11b- APC (clone D12; BD). Subsequently, 30 min of incubation at room temperature erythrocytes were lysed using 2 ml of FACS lysing solution (BD Biosciences Pharmingen), cells were washed twice with 2 ml of PBS and suspended in 200ul of PBS (Lonza, Walkersville, MD). Eight- colour flow cytometry was done on a FACSCantoII flow cytometer with FACSDIVA software, version 6 (Becton Dickinson). The gating was set by forward and side scatter, and 1,00,000 gated events were acquired. Data were collected and analysed using FLOW JO software (TreeStar, Ashland, OR). CD45 expression versus side scatter gating strategy was used to gate the Leukocytes.

### Statistical analysis

Based on preliminary analysis, a sample size of 30 participants per arm was determined to be sufficient to detect a cell count difference of 26 with 90% power at the 5% level of significance assuming a pooled standard deviation of 5 to detect differences in the absolute counts of T, B and NK cells. Central tendency was measured using Geometric means (GM). Statistically significant differences between two groups were analysed using non-parametric Mann–Whitney U-test. Multiple comparisons for each set of experiments (T cell subsets; B cell subsets; monocyte subsets and dendritic cell subsets) were individually corrected using the Holm’s correction. Thus, p values obtained by the Mann-Whitney U test for each cell subset were corrected by recalculating them using the Holm’s correction method. Data analyses were performed using GRAPHPAD PRISM Version 6 (GraphPad, San Diego, CA). The Spearman rank correlation matrix analysis was done using JMP 13 (SAS) software.

## Results

### Study population characteristics

Based on BMI and serum albumin, LTBI positive individuals were categorized into two groups: (i) LBMI with BMI of <18.5 kg/m^2^ and serum albumin levels of <3.4 g/dl and (ii) NBMI with BMI between 18.5 and 24.9 kg/m^2^ and serum albumin levels between 3.7 to 4.8 g/dl. There was no statistically significant difference between the two groups in the levels of random blood glucose, HbA1c, urea, creatinine, ALT and AST levels. The haematological parameters are shown in Table 2. The haematological parameters did not show any significant difference between the two groups. Age or gender did not differ significantly between two groups.

**Table 2:** Haematological parameters of the study population.

Hematological Parameters	LBMI (n = 30)	NBMI (n = 30)	p value
Hb gm/dL GM (Range)	12.4 (9.2–15.1)	13.4 (10.2–18.6)	0.1440
RBC 10^6^/mL GM (Range)	4.6 (3.5–5.3)	4.8 (3.9–6.4)	0.2943
WBC 10^3^/mL GM (Range)	7.1 (4.6–11.5)	7.4 (4.1–12)	0.1000
HCT % GM (Range)	38.4 (19.6–52.4)	41.1 (24.4–58.6)	0.9191
PLT 10^3^/mL GM (Range)	248.9 (124–372)	265.5 (137–390)	0.3356
Neutrophil 10^3^/mL GM (Range)	3.57 (1.5–5.6)	3.7 (2.5–6.9)	0.5950
Lymphocyte 10^3^/mL GM (Range)	2.34 (1.67–3.12)	2.6 (1.96–4.2)	0.8652
Monocyte 10^3^/mL GM (Range)	6.2 (2–8.3)	6.8 (4–9.5)	0.2650
Eosinophil 10^3^/mL GM (Range)	6.97 (1.3–26.3)	5.1 (1.2–13)	0.5438
Basophil 10^3^/mL GM (Range)	0.71 (0.2–2.1)	0.89 (0.6–5.8)	0.8747

### Low BMI is associated with diminished frequencies and absolute counts of T cells, B cells and NK cells

**Fig 1:**
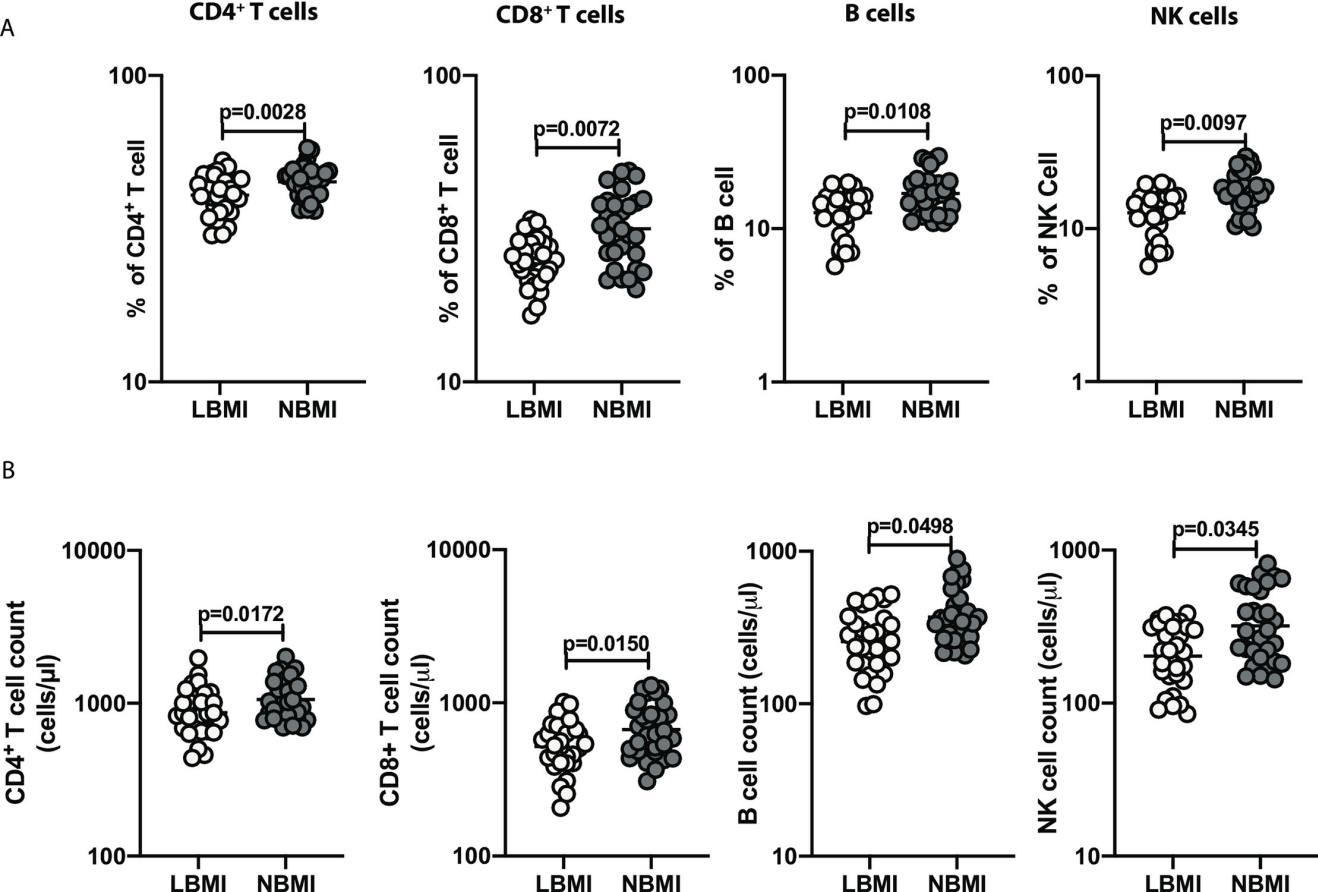
Low BMI is associated with diminished frequencies and absolute counts of T cells, B cells and NK cells. (A) The frequencies of CD4^+^ T cells, CD8^+^ T cells, B cells and NK cells in LTBI-LBMI [LBMI] (n = 30) or LTBI-NBMI [NBMI] (n = 30) group were measured by flow cytometry clinical software. (B) The absolute counts of CD4^+^ T cells, CD8^+^ T cells, B cells and NK cells in LTBI-LBMI [LBMI] (n = 30) or LTBI-NBMI [NBMI] (n = 30) group were measured by flow cytometry clinical software. The data are depicted as scatter plots and each circle represent a single person. Mann–Whitney U-test with Holms correction for multiple comparisons were done to calculate p values.

To study the effect of BMI on leukocyte subsets in LTBI, we determined the frequencies and cell count of T cells, B cells and NK cells in LBMI and NBMI individuals with coexistent LTBI. As can be seen in Fig 1A, the frequencies of CD4^+^ T cells (Geometric mean (GM) of 40.7% in LBMI versus 44.99% in NBMI, p = 0.0028), CD8^+^ T cells (GM of 24.99% in LBMI versus 31.6% in NBMI, p = 0.0072), B cells (GM of 12.72% in LBMI versus 16.98% in NBMI, p = 0.0108) and NK cells (GM of 12.5% in LBMI versus 17.74% in NBMI, p = 0.0097) were significantly lower in the LBMI group when compared with the NBMI group. As can be seen in Fig 1B, the LBMI group exhibited significantly diminished cell counts of CD4^+^ T cells (GM of 869.7 cells/μl in LBMI versus 1060 cells/μl in NBMI, p = 0.0172), CD8^+^ T cells (GM of 519.2 cells/μl in LBMI versus 669.2 cells/μl in NBMI, p = 0.0150), B cells (GM of 255.4 cells/μl in LBMI versus 371.4 cells/μl in NBMI, p = 0.0498) and NK cells (GM of 202.8 cells/μl in LBMI versus 318.7 cells/μl in NBMI, p = 0.0345) in comparison with NBMI group. Unlike our hematology data, the more sensitive FACS Trucount technique was able to detect differences in lymphocyte numbers between the groups. Thus, LBMI with LTBI is associated with decreased frequencies and cell counts of T cells, B cells and NK cells.

### LBMI is associated with altered frequencies of CD4^+^ T cells and CD8^+^ T cells

**Fig 2:**
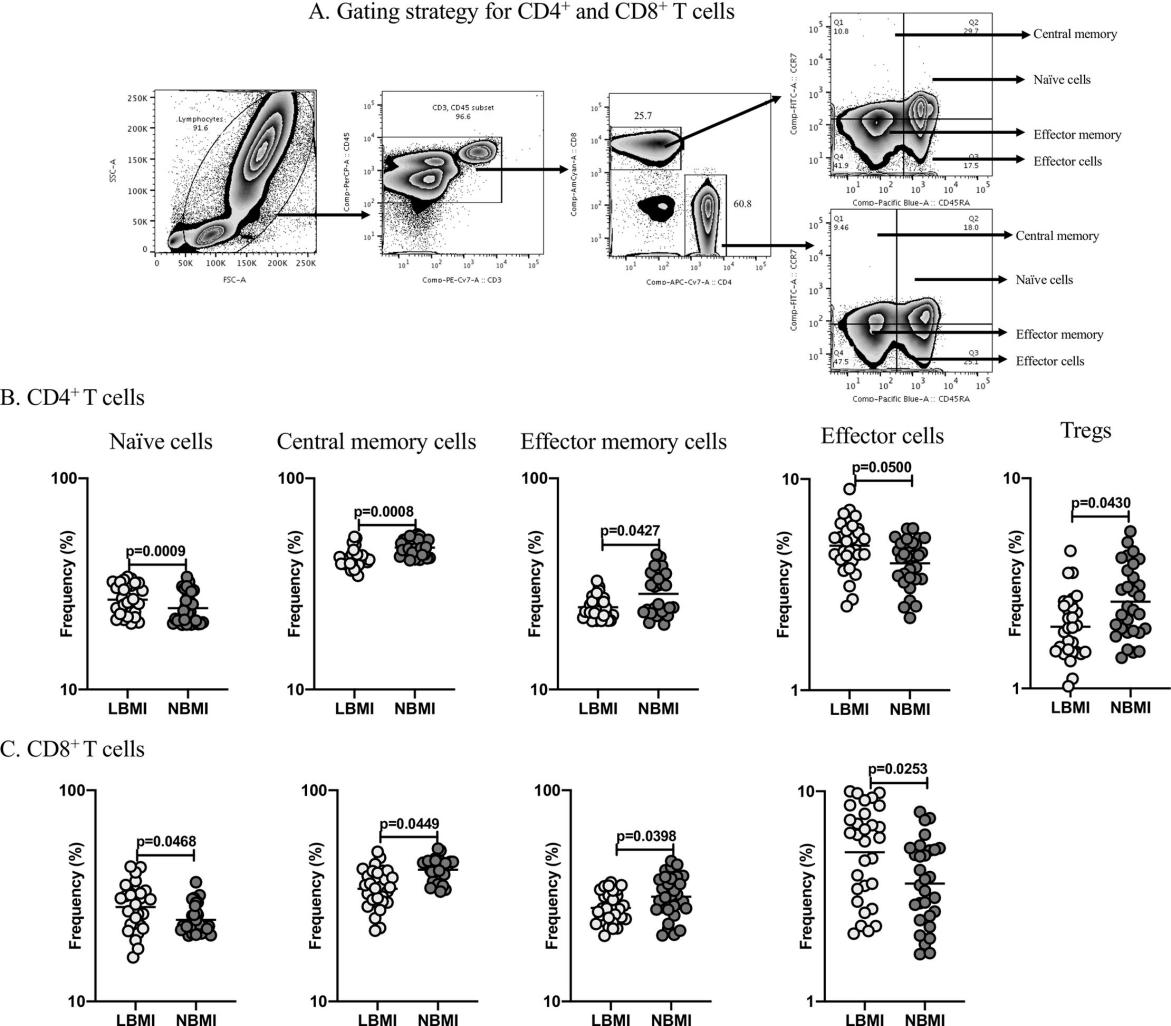
LBMI is associated with altered frequencies of CD4^+^ T cells and CD8^+^ T cells. **(**A) Gating strategy for CD4^+^ T cells and CD8^+^ T cells: An illustrative flow cytometry plot from a LTBI-LBMI individual depicting the gating strategy for naïve, central memory and effector memory cells from CD4^+^ and CD8^+^ T cells. Naïve cells were defined by the expression of CD45RA^+^ CCR7^+^; effector memory cells as CD45RA^-^ CCR7^-^; central memory cells as CD45RA^-^ CCR7^+^; and effector cells as CD45RA^+^CCR7^-^. (B) The frequencies of CD4^+^ T cell subsets—naïve cells, central memory, effector memory cells, effector cells and regulatory T cells in LTBI-LBMI [LBMI] (n = 30) or LTBI-NBMI [NBMI] (n = 30) group. (C) The frequencies of CD8^+^ T cell subsets—naïve cells, central memory, effector memory cells, effector cells and regulatory T cells in LTBI-LBMI [LBMI] (n = 30) or LTBI-NBMI [NBMI] (n = 30) group. The data are depicted as scatter plots and each circle represent a single person. Mann–Whitney U-test with Holms correction for multiple comparisons were done to calculate p values.

Next, to determine the influence of BMI on CD4^+^ T cell and CD8^+^ T cell subsets in LTBI, we measured the frequencies of different CD4^+^ T and CD8^+^ T cell subsets (naive, central memory, effector memory, effector and regulatory) in LBMI and NBMI groups. Fig 2A depicts an illustrative flow cytometry dot plot of CD4^+^ T cells and CD8^+^ T cells gating strategy. As can be seen in Fig 2B, LBMI group exhibited significantly enhanced frequencies of naïve (GM of 26.66% in LBMI versus 24.29% in NBMI, p = 0.0009) and effector CD4^+^ T cells (GM of 4.83% in LBMI versus 3.99% in NBMI, p = 0.0500) when compared with NBMI group. In contrast, the frequencies of central memory (GM of 41.66% in LBMI versus 46.97% in NBMI, p = 0.0008), effector memory (GM of 24.51% in LBMI versus 28.35% in NBMI, p = 0.0427) and regulatory CD4^+^ T cells (GM of 1.97% in LBMI versus 2.59% in NBMI, p = 0.0430) were significantly diminished when compared with NBMI group. S2A Fig depicts an illustrative flow cytometry dot plot of regulatory T cell gating strategy. As can be seen in Fig 2C, LBMI group showed significantly increased frequencies of naïve cells (GM of 27.99% in LBMI versus 24.32% in NBMI, p = 0.0468) and effector CD8^+^ T cells (GM of 5.13% in LBMI versus 3.65% in NBMI, p = 0.0253) when compared with NBMI group. In contrast, the frequencies of central memory (GM of 34.19% in LBMI versus 42.05% in NBMI, p = 0.0449) and effector memory CD8+ T cells (GM of 27.72% in LBMI versus 31.34% in NBMI, p = 0.0398) were significantly diminished when compared with NBMI group. Therefore, LBMI with LTBI is associated by changes in the CD4^+^ and CD8^+^ T cell subset frequencies distribution.

### LBMI is associated with diminished frequencies of B cell subsets

**Fig 3:**
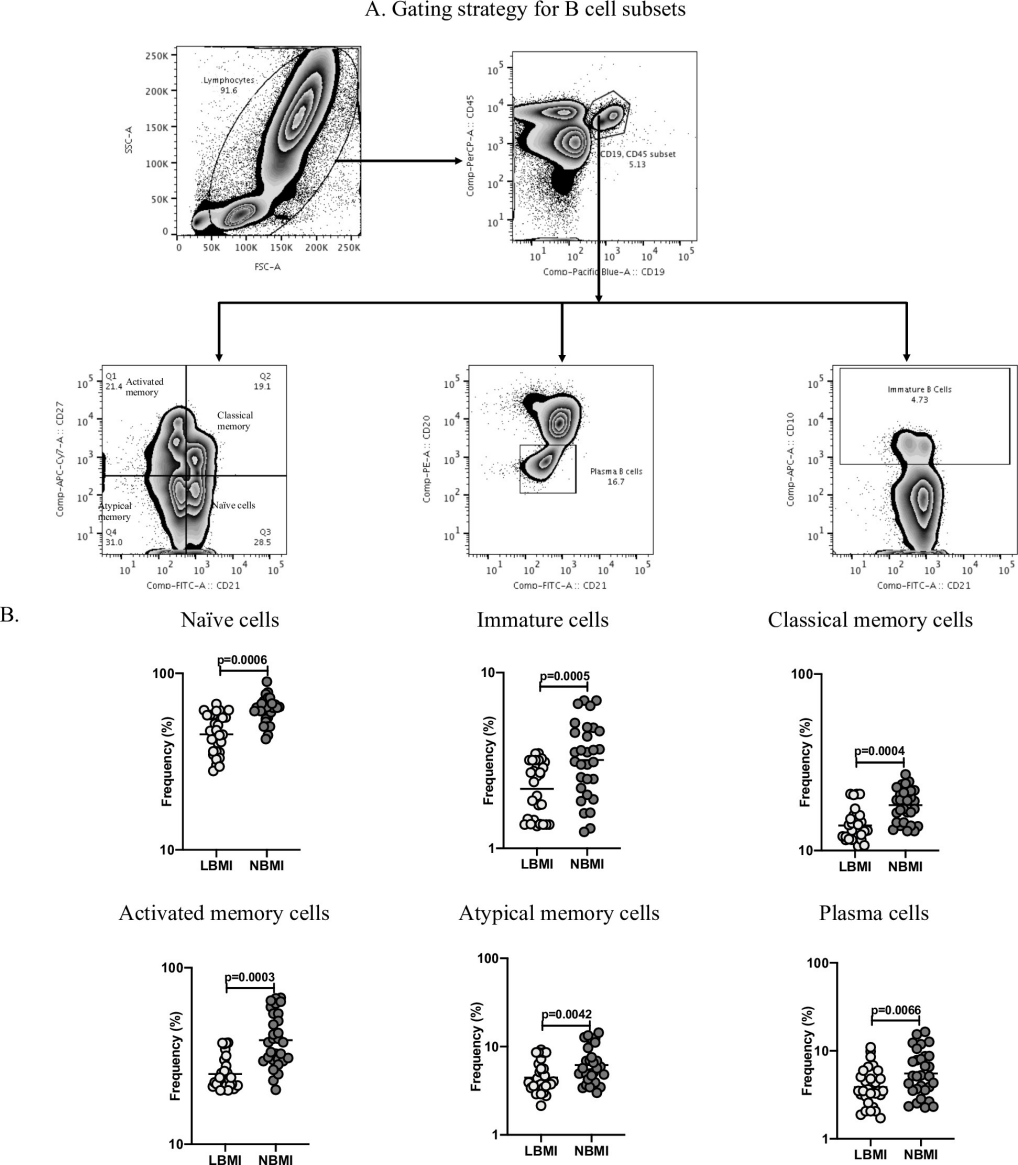
LBMI is associated with diminished frequencies of B cell subsets. (A) Gating strategy for B cell subsets: An illustrative flow cytometry plot from an LTBI-LBMI individual depicting the gating strategy for naïve, immature, classical memory (CM), activated memory (AM), atypical memory (ATM), immature and plasma cells from CD45^+^ CD19^+^ cells. Naïve cells were classified as CD21^+^ CD27^-^; classical memory (CM) cells as CD21^+^ CD27^+^; activated memory (AM) cells as CD21^-^ CD27^+^; Atypical memory (ATM) cell as CD21^-^ CD27; immature B cells as CD21^+^ CD10^+^; and plasma cells as CD21^-^ CD27^-^. (B) Frequencies of B cell subsets—naïve B cells, classical memory B cells (CM), activated memory (AM), atypical memory (ATM), immature and plasma cells in LTBI-LBMI [LBMI] (n = 30) or LTBI-NBMI [NBMI] (n = 30) group. The data are depicted as scatter plots and each circle represent a single person. Mann–Whitney U-test with Holms correction for multiple comparisons were done to calculate p values.

Subsequently, to study the effect of BMI on B cell (and B cell subset) phenotype in LTBI, we measured the frequencies of B cell subsets (naïve cells, immature cells, classical memory cells, activated memory cells, atypical memory cells and plasma cells) in LBMI and NBMI individuals. Fig 3A depicts an illustrative flow cytometry dot plot of B cell subsets gating strategy. As can be seen in Fig 3B, LBMI group showed significantly diminished frequencies of naïve B cells (GM of 45.18% in LBMI versus 62.2% in NBMI, p = 0.0006), immature B cells (GM of 2.18% in LBMI versus 3.18% in NBMI, p = 0.0005), classical memory B cells (GM of 13.86% in LBMI versus 18.08% in NBMI, p = 0.0004), activated memory B cells (GM of 24.99% in LBMI versus 38.86% in NBMI, p = 0.0003), atypical memory B cells (GM of 4.46% in LBMI versus 6.18% in NBMI, p = 0.0042) and plasma cells (GM of 3.87% in LBMI versus 5.52% in NBMI, p = 0.0066) when compared with NBMI group. Therefore, LBMI with LTBI is associated with decreased frequencies of B cell subsets.

### LBMI is associated with altered frequencies of monocyte and dendritic cell subsets

**Fig 4:**
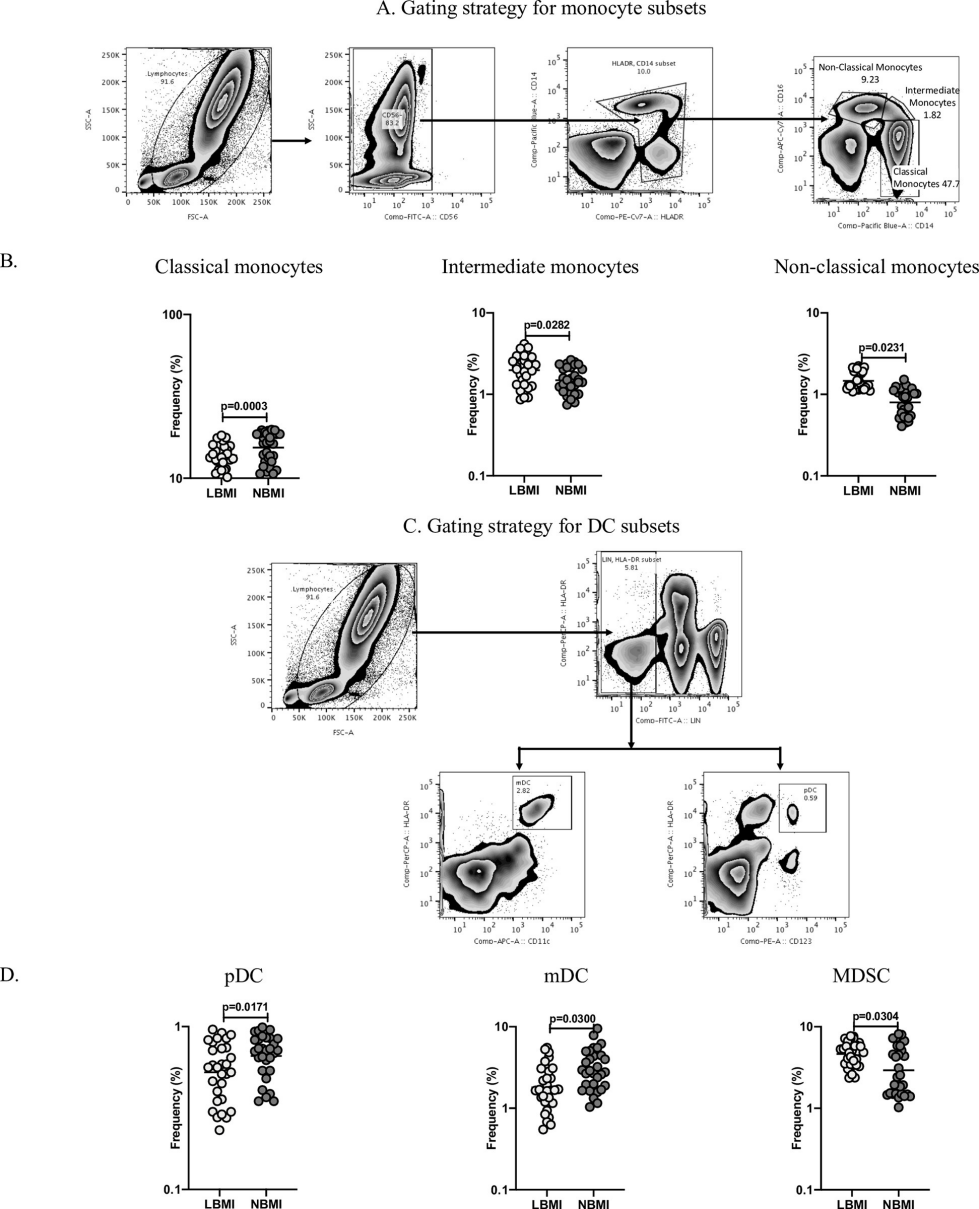
LBMI is associated with altered frequencies of monocyte and dendritic cell subsets. (A) Gating strategy for monocyte subsets: An illustrative flow cytometry plot depicting the gating strategy for monocyte subsets. Classical monocytes were defined by the expression of CD45^+^ HLA-DR^+^ CD14^hi^ CD16^–^; intermediate monocytes as CD45^+^ HLA-DR^+^ CD14^hi^ CD16^dim^ and non-classical monocytes were defined by the expression of CD45^+^ HLADR^+^ CD14^dim^ CD16^hi^. (B) The frequencies of monocyte (classical, intermediate and non-classical) subsets in LTBI-LBMI [LBMI] (n = 30) or LTBI-NBMI [NBMI] (n = 30) individuals. (C) An illustrative flow cytometry plot from an LTBI-LBMI individual depicting the gating strategy of plasmacytoid (pDC) and myeloid DCs (mDC). Plasmacytoid DC were defined by the expression of (Lin^−^HLA-DR^+^ CD123^+^) and myeloid DCs as (Lin^−^HLA-DR^+^ CD11c^+^). (D) The frequencies of DC (pDC, mDC and MDSC) subsets in LTBI-LBMI [LBMI] (n = 30) or LTBI-NBMI [NBMI] (n = 30) group. The data are depicted as scatter plots and each circle represent a single person. Mann–Whitney U-test with Holms correction for multiple comparisons were done to calculate p values.

Next, to determine the influence of BMI on monocyte and dendritic cell phenotype in LTBI, we first measured the frequencies of monocyte subsets (classical monocytes, intermediate monocytes and non-classical monocytes) in LBMI and NBMI groups. Fig 4A depicts an illustrative flow cytometry dot plot of monocyte subsets gating strategy. As can be seen in Fig 4B, LBMI group showed significantly diminished frequencies of classical monocytes (GM of 13.78% in LBMI versus 15.39% in NBMI, p = 0.0003) when compared with NBMI group. In contrast, LBMI group exhibited significantly enhanced frequencies of intermediate monocytes (GM of 1.97% in LBMI versus 1.49% in NBMI, p = 0.0282) and non-classical monocytes (GM of 1.46% in LBMI versus 0.8% in NBMI, p = 0.0231) in comparison with NBMI individuals. Further, we examined the frequencies of dendritic cell subsets (myeloid DCs and plasmacytoid DCs) in LBMI and NBMI individuals. Fig 4C depicts an illustrative flow cytometry dot plot of dendritic cell subsets gating strategy. As can be seen in Fig 4D, LBMI group showed significantly decreased frequencies of plasmacytoid DCs (GM of 0.52% in LBMI versus 0.66% in NBMI, p = 0.0171) and myeloid DCs (GM of 1.80% in LBMI versus 2.94% in NBMI, p = 0.0300) when compared with NBMI group. Finally, we examined the frequencies of MDSC in LBMI and NBMI groups. S2B Fig depicts an illustrative flow cytometry dot plot of MDSC gating strategy. LBMI group exhibited significantly enhanced frequencies of MDSC (GM of 4.64% in LBMI versus 2.92% in NBMI, p = 0.0304) when compared with NBMI group. Therefore, LBMI with LTBI is associated with altered frequencies of monocyte and dendritic cell subsets.

### Relationship between immune cells and BMI

**Fig 5:**
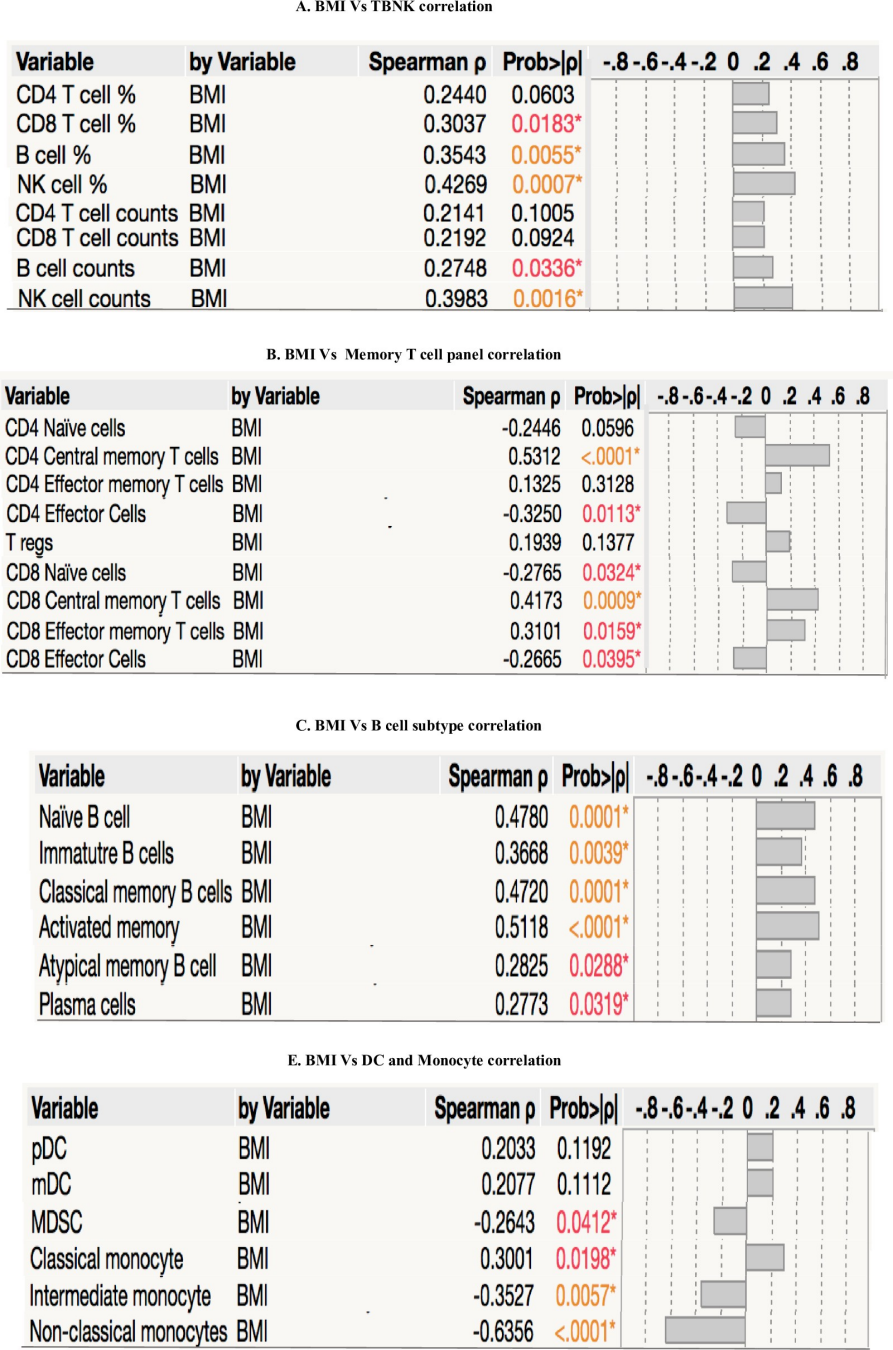
Relationship between immune cells and BMI. Relationship between immune cell frequencies and BMI in LTBI individuals. The relationship between the (A) Frequencies and cell counts of CD4^+^ T cells, CD8^+^ T cells, B cells and NK cells and BMI were examined in all individuals with LTBI (n = 60). (B) Frequencies of CD4^+^ T cells, CD8^+^ memory T cells and BMI were assessed in LTBI (n = 60) (C) Frequencies of B cell subsets and BMI were assessed in LTBI (n = 60) (D) Frequencies of monocyte and DC subsets and BMI were assessed in LTBI (n = 60). The data are depicted as correlation rank matrices with r values being indicated by horizontal bars. p and r values were determined by Spearman rank correlation test at 95% confidence intervals by JMP software.

Next, we determined the association between T cell-, B cell-, monocyte- and dendritic cell (DC)- subset frequencies and BMI. As shown in Fig 5, the frequencies of B cell and B cell subsets, NK cells, central memory T cells (CD4^+^ and CD8^+^ T cells), effector memory T cells (CD8^+^ T cells) and classical monocyte exhibited significant positive correlation with BMI. In contrast, the frequencies of naïve cells (CD4^+^ cells), effector cells (CD4^+^ and CD8^+^ T cells), intermediate monocytes, non-classical monocytes and myeloid derived suppressor cells exhibited significant negative association with BMI.

## Discussion

Even though nutrition is documented as being an important element of immunity, the mechanism by which undernutrition affects the immune functions remains unclear [13, 14]. Both innate and adaptive immune systems are influenced by the nutritional status. Animal studies revealed that diminished T cell numbers were observed in severe undernutrition or starvation of animal models, which also affect T‐cell survival and proliferation [15, 16]. Both CD4^+^ and CD8^+^ T cell counts were diminished in undernourished humans when compared with normal weight controls [17]. A previous study showed that undernourished individuals exhibited diminished CD4/CD8 ratio and increased numbers of CD4 and CD8 double negative T cells [18]. Schlesinger *et al*. reported that undernourished mice had reduced NK cell numbers and NK cell activity [19]. In corroboration with this, our data also reveal diminished levels of CD4^+^, CD8^+^ T cell, B cell and NK cell frequencies as well as counts in LBMI individuals with LTBI. Frequencies and counts of T cells, B cell and NK cells were significantly positively correlated with BMI. Thus, low BMI induces alteration in cell-mediated immunity which could potentially affect individuals host response to infection.

In humans, the association between undernutrition and homeostatic memory cell maintenance is unknown. Memory cells are critical for the defence from reinfection. The defence mechanism depends on antigen-independent persistence of memory T and B cells primed to respond rapidly upon re-challenge. Memory homeostasis process involves cell survival which relies on nutrient availability [20, 21]. There is a paucity of information on how nutrition influences memory T cell homeostasis. Proliferation of memory cells requires dietary protein which might be crucial for maintaining memory T cells. Studies on protein energy malnutrition (PEM) mice models showed that lymphocytic choriomeningitis virus (LCMV)-specific CD8^+^ T_EM_ were diminished and T_EM_ were not sustained and in tuberculosis model exhibited reduced lung CD4^+^ T cell responses to mycobacterial antigens [22, 23]. It has also been shown that in undernourished animals, nutritional replenishment resulted in reconstitution of memory T cell responses which improves pathogen clearance [22, 23]. A study on undernourished mice revealed that there was a significant and swift reduction in the number of thymocytes, splenocytes, and lymphocytes in these mice [24]. More recent data on undernourished mice demonstrated that mice exhibited significant decreased numbers of effector and regulatory T cells and also alteration in the balance of T cell subsets [25, 26]. Our data also reveal that significantly diminished frequencies of classical memory, effector memory and regulatory T cell subsets are characteristics of LBMI with LTBI. BMI exhibited negative correlation with naive and effector T cells, while central memory and effector memory T cells exhibited significant positive correlation with BMI. Therefore, our data reveal that low BMI is associated with altered frequencies of memory T cell subsets in LTBI, perhaps enhancing the possibility of progression to active TB disease.

While there is an increasing indication of the importance B cell responses to intracellular pathogens, B cell subsets in TB have not been studied in detail [27]. Studies have shown that both naive and memory B cells are present in tuberculosis granulomas [27]. *M*.*tb*-infected lung contains B cells, which may be involved in the process of *M*.*tb* antigen presentation to T cells and secrete cytokines and *M*.*tb*-specific antibodies [28]. In our study, LBMI group showed significantly decreased percentages of B cell subsets and also the frequencies of B cells exhibited a significant positive correlation with BMI. Mice deficient in B cells are more vulnerable to *M*.*tb* infection and have impaired capability to produce *M*.*tb* specific antibodies [29, 30]. Abe *et al* reported that undernourished mice exhibited decreased levels of B lymphocytes and these B cell subsets have impairment in the secretion of immunoglobulin, cytokine and proliferation [31–33]. Our data on diminished frequencies of classical memory B cells, activated memory B cells and plasma cells suggests that the capacity of B cells to secrete antibodies in the face of ongoing TB infection might be compromised in these individuals. Our data also suggest that undernutrition impairs the capacity of B cell production, maintenance or proliferation since, all the subsets of B cells are decreased in frequency.

Monocytes are the major players in the immune response against TB. Based on the relative expression of CD14 and CD16, the monocyte subsets are divided into classical, intermediate and non-classical monocytes [34]. Classical monocytes consist of about 80–95% of circulating monocytes, known to be main scavenger cells and are highly phagocytic. Intermediate monocytes encompass up to 5% of total monocytes. These subsets plays a role in production of reactive oxygen species (ROS), antigen presentation and are also involved in T cell proliferation and stimulation and inflammatory responses [35, 36]. Non-classical monocytes consist of 5–10% of monocytes. These subsets are known as patrolling monocytes and are pro-inflammatory in behaviour and produce inflammatory cytokines in response to infection [36, 37]. Our findings reveal that intermediate and non-classical monocyte percentage was significantly decreased in LBMI compared to NBMI group. Moreover, BMI exhibited negative correlation with frequencies of monocyte subsets. The innate immune response functions such as phagocytosis and secretion of reactive oxygen and nitrogen intermediates by macrophages are affected due to undernutrition [31]. Recent studies have reported that *M*.*tb* infection alters the CD16 intermediate monocyte subset, implying that this subset supports the intracellular persistence of *M*.*tb* [38]. The altered frequencies of monocyte subsets suggest that LTBI with LBMI group are associated with defect in their monocyte functions.

DCs plays the major role in bridging innate and adaptive immune response via their function in encapsulating, processing and presenting antigens. DCs have been categorized as HLADR^+^ cells and based on expression classified into myeloid DCs (CD11c^+^) or plasmacytoid DCs (CD123^+^) [39]. Earlier studies stated that migration of DC to the draining lymph node is necessary for the triggering naive T cells in TB infection [40]. Myeloid DCs and plasmacytoid DCs plays key role in stimulation of CD4^+^ and CD8^+^ T cells and support the antibacterial activity [41, 42]. *M*.*tb*-infected mice showed worsening of disease and enhanced survival of *M*.*tb* due to excessive deposition of myeloid-derived suppressor cells (MDSC) [43]. Several studies have stated the negative role of MDSC in anti-TB immunity, T cell activation, proliferation, trafficking, regulatory T cell stimulation and T cell cytokine responses [44–46]. Our data reveals that the percentage of plasmacytoid and myeloid DC are significantly diminished and in contrast, MDSC frequencies were significantly higher in LBMI in comparison with NBMI group. The functional effects of this diminished phenotypic distribution of DC subsets in LTBI-LBMI individuals requires additional studies. Previous studies have shown that diminished frequencies of DC subsets could lead to impaired production of IL-12, fewer IFNγ T cells and lower T cells proliferation due to undernutrition [31, 47]. Our data therefore indicate that low BMI has profound effects on the DC subsets in LTBI.

Our data offers an understanding into the effect of nutrition on the pathogenesis of TB. To our knowledge, this is the first study to examine the impact of low BMI on innate and adaptive immune cells subsets in the context of LTBI. Although, our data do not provide any mechanistic conclusions, our data offers an important preliminary substantiation for a major relationship between undernutrition and immune cell composition in a chronic infection. Our study suffers from the limitation of not being a longitudinal study, of not measuring micronutrient levels or body fat composition and of having a modest sample size. Also, the cellular analysis is non-specific and these are not mycobacterial–antigen stimulated cells. Longitudinal studies of low BMI individuals with LTBI should help elucidate the mechanism by which undernutrition promotes the progression from latent to active infection. Nevertheless, our study clearly implies that perturbations in immune cell frequencies and numbers might be involved in the process. In conclusion, by studying the innate and adaptive cellular responses at homeostasis in undernourished individuals with LTBI, our study offers the direct relationship of compromised immune cellular response in individuals with low BMI and LTBI.

## Supporting information

S1 Fig Flowchart for the study sample recruitment. A flowchart illustrating the numbers of samples recruited to the study.
(TIF)

S2 Fig Gating strategy for regulatory T cells and myeloid-derived suppressor cells (MDSC). (A) An illustrative flow cytometry plot depicting the gating strategy of regulatory T cells. Tregs were defined by the expression of CD4^+^, CD25^+^, Foxp3^+^, CD127^dim^. (B) An illustrative flow cytometry plot depicting the gating strategy of myeloid-derived suppressor cells **(**MDSC). MDSCs were defined by the expression of CD45^+^,CD33^+^, HLA-DR^-^ CD11b^+^.
(TIF)

S1 File Dataset. (XLSX)
