# Supplementary figures and images for: Undernutrition is associated with perturbations in T cell-, B cell-, monocyte- and dendritic cell- subsets in latent Mycobacterium tuberculosis infection
Source: PLoS One. 2019 Dec 10;14(12):e0225611. doi: 10.1371/journal.pone.0225611 (PMC6903744; doi:10.1371/journal.pone.0225611)

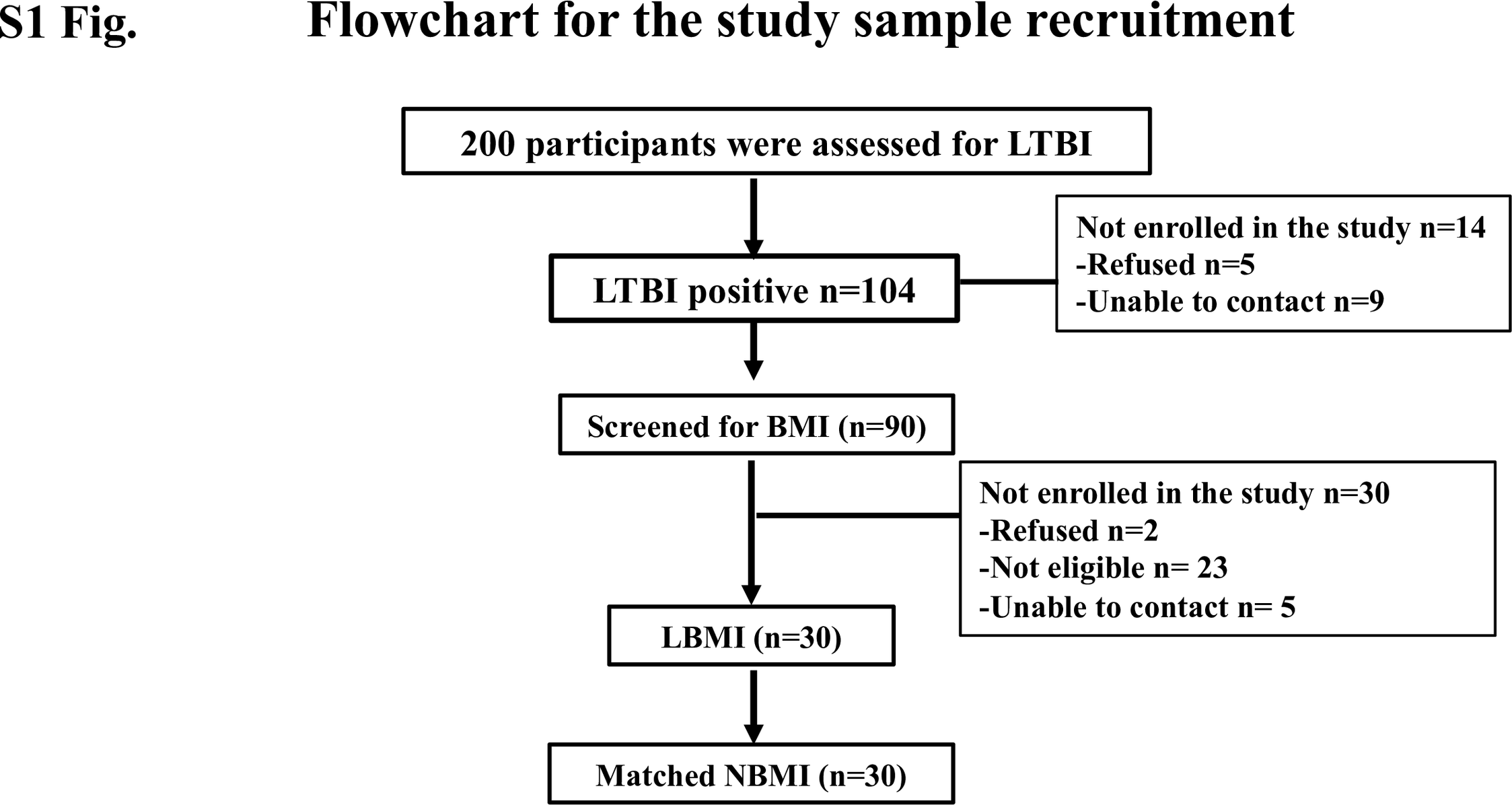

Supplement: S1 Fig — A flowchart illustrating the numbers of samples recruited to the study. (TIF) [file pone.0225611.s001.tif]

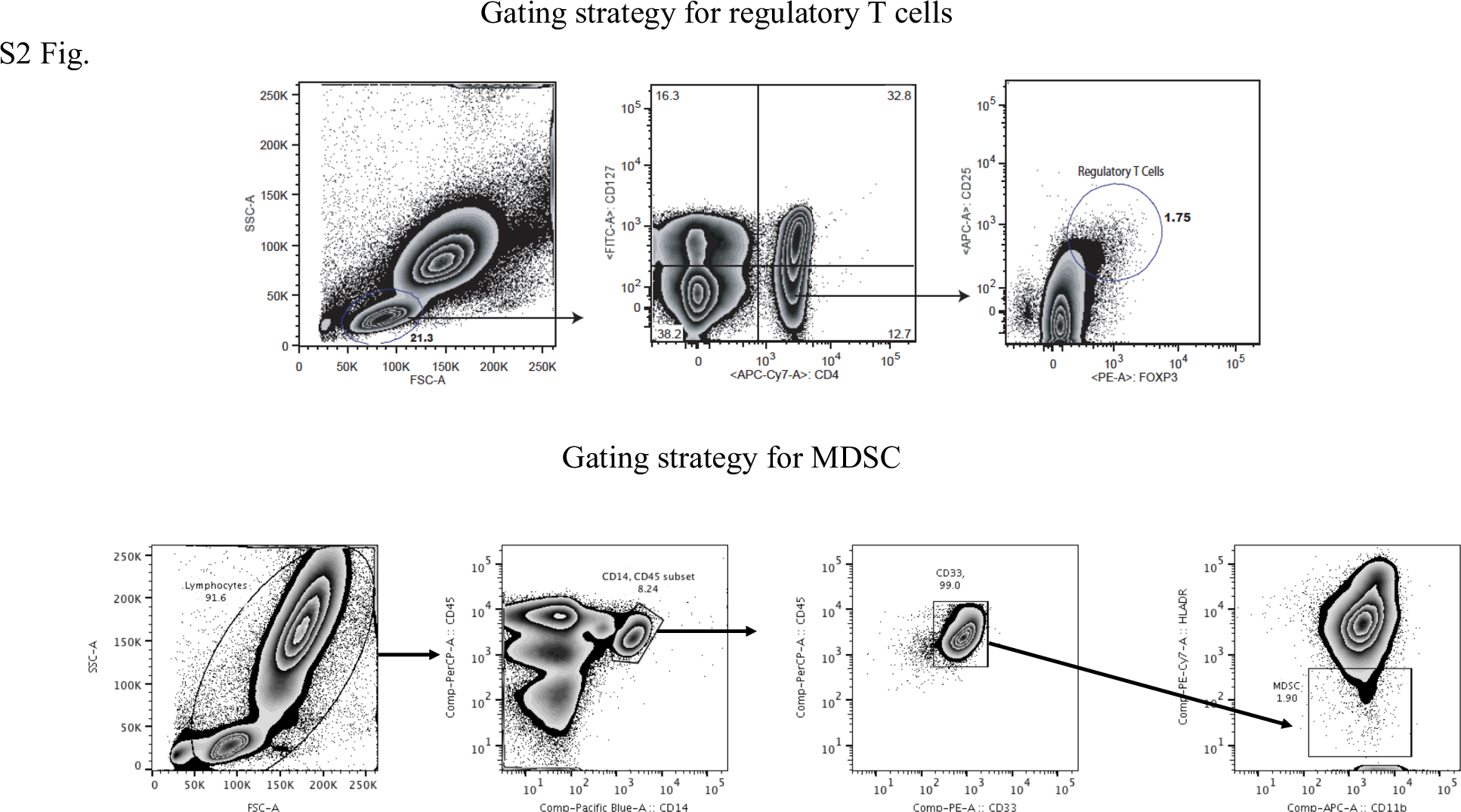

Supplement: S2 Fig — (A) An illustrative flow cytometry plot depicting the gating strategy of regulatory T cells. Tregs were defined by the expression of CD4+, CD25+, Foxp3+, CD127dim. (B) An illustrative flow cytometry plot depicting the gating strategy of myeloid-derived suppressor cells (MDSC). MDSCs were defined by the expression of CD45+,CD33+, HLA-DR- CD11b+. (TIF) [file pone.0225611.s002.tif]
